# Damage-associated molecular patterns in hepatic ischemia–reperfusion injury: spatiotemporal signatures, biomarker potential, and clinical translation

**DOI:** 10.3389/fimmu.2026.1789287

**Published:** 2026-05-28

**Authors:** Peng An, Yi An, Mengwei Chen, Longlong Wu, Rong Wang

**Affiliations:** 1 The Gastroenterology Department of Shanxi Provincial People’s Hospital, Shanxi Medical University, Taiyuan, China; 2 Taiyuan Peace Hospital, Taiyuan, China

**Keywords:** Biomarkers, clinical translation, damage-associated molecular patterns, hepatic ischemia–reperfusion injury, machine perfusion, spatiotemporal signatures

## Abstract

Hepatic ischemia–reperfusion injury (HIRI) remains a key contributor to early allograft dysfunction (EAD), post-hepatectomy liver failure, biliary complications, and the underutilization of marginal donor grafts. Damage-associated molecular patterns (DAMPs) are central mediators of sterile inflammation in HIRI; however, their translational relevance extends beyond being upstream inflammatory triggers. In this review, we reframe DAMP biology by considering spatiotemporal release, sampling accessibility, biomarker utility, and therapeutic tractability. Rather than providing another broad overview of sterile inflammation or regulated cell death, we focus on how distinct DAMP classes—including nuclear and nucleic acid-related signals, mitochondrial and metabolic DAMPs, extracellular matrix-derived fragments, and protein-based danger cues—arise from defined cellular sources during cold ischemia, warm ischemia, early reperfusion, and late injury–repair phases. We discuss how these signals are detected by pattern-recognition receptors (PRRs) and intracellular sensing pathways, including Toll-like receptors (TLRs), receptor for advanced glycation end products (RAGE), the P2X purinoceptor 7 (P2X7)–NLR family pyrin domain-containing 3 (NLRP3) inflammasome axis, and cyclic GMP–AMP synthase (cGAS)–stimulator of interferon genes (STING) signaling. We further examine how DAMP-driven sensing coordinates Kupffer cell activation, endothelial dysfunction, neutrophil-mediated amplification, thromboinflammation, and impaired tissue recovery. Particular emphasis is placed on circulating and perfusate-accessible DAMP signatures as minimally invasive tools for injury grading, EAD prediction, biliary complication risk stratification, and donor graft-quality assessment during machine perfusion. We also summarize DAMP-targeted therapeutic strategies, including strategies to limit DAMP release, enhance extracellular DAMP clearance, block dominant sensing pathways, and integrate DAMP modulation with organ-preservation platforms. Finally, we highlight key barriers to clinical translation, including assay heterogeneity, undefined sampling windows, insufficient multicenter validation, and the need to distinguish reparative from pathogenic DAMP signaling. A spatiotemporal, DAMP-centered framework may help connect molecular injury biology with biomarker-guided decision-making and precision interventions in liver transplantation and hepatic surgery.

## Graphical Abstract

**Figure:**
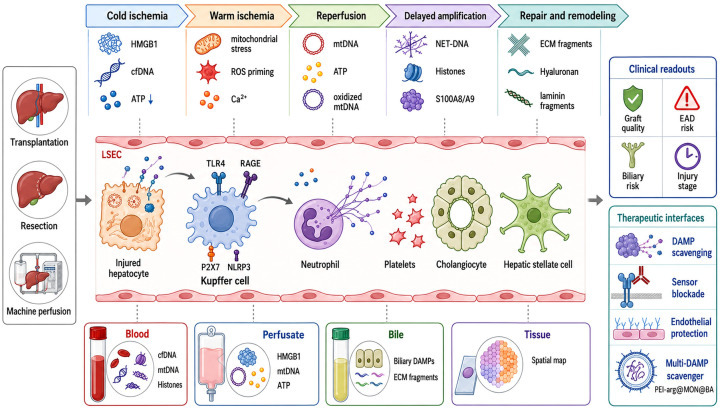

## 1 Introduction

Hepatic ischemia–reperfusion injury (HIRI) is a clinically significant and mechanistically complex process that can occur during liver transplantation, hepatic resection with vascular inflow occlusion, traumatic shock, and other conditions characterized by transient interruption and subsequent restoration of hepatic blood flow. In liver transplantation, donor grafts undergo cold ischemia during preservation, warm ischemia during implantation, and abrupt metabolic reactivation after reperfusion (1). In hepatic surgery, regional ischemia is often intentionally induced to reduce intraoperative blood loss. Although reperfusion is essential for tissue survival, it can paradoxically exacerbate hepatocellular injury, liver sinusoidal endothelial dysfunction, microcirculatory disturbance, biliary injury, and postoperative liver dysfunction (2). Clinically, HIRI contributes to early allograft dysfunction (EAD), primary graft non-function, ischemic biliary complications, acute and chronic rejection, prolonged hospitalization, and underutilization of marginal donor grafts, particularly steatotic grafts, grafts from elderly donors, and donation-after-circulatory-death (DCD) livers (3).

The pathogenesis of HIRI cannot be explained by ischemic hypoxia alone. Ischemia induces adenosine triphosphate (ATP) depletion, mitochondrial depolarization, ionic imbalance, metabolic stress, and impaired antioxidant capacity. Reperfusion then converts these intracellular disturbances into oxidative stress, innate immune activation, vascular injury, and amplification of regulated cell death (3, 4). Hepatocytes, liver sinusoidal endothelial cells (LSECs), Kupffer cells (KCs), recruited monocyte-derived macrophages, neutrophils, platelets, cholangiocytes, and hepatic stellate cells collectively shape this injury response. In the early reperfusion phase, reactive oxygen species (ROS) generation, calcium overload, mitochondrial permeability transition, and lipid peroxidation promote organelle disruption and hepatocyte death. Emerging mechanistic studies further suggest that calcium influx during reperfusion, arachidonic acid metabolism, and lipid peroxidation converge to increase ferroptotic susceptibility, particularly in metabolically stressed or steatotic grafts. These findings support a dynamic model in which metabolic failure, mitochondrial injury, sterile inflammation, microvascular dysfunction, and cell death mutually reinforce each other rather than acting as isolated processes (5).

Damage-associated molecular patterns (DAMPs) provide a critical molecular bridge between cellular injury and sterile inflammatory activation in HIRI. DAMPs are endogenous molecules that are normally sequestered within intracellular compartments, mitochondria, nuclei, granules, or the extracellular matrix, but acquire immunostimulatory properties upon release, exposure, oxidation, fragmentation, or redistribution during tissue stress (6). In the ischemic and reperfused liver, representative DAMPs include high-mobility group box 1 (HMGB1), extracellular histones, cell-free DNA (cfDNA), mitochondrial DNA (mtDNA), ATP, S100A8/A9, heat shock proteins, lipid peroxidation-derived mediators, and extracellular matrix fragments (7–9). These molecules are sensed by cell-surface and intracellular pattern-recognition receptors (PRRs), including Toll-like receptors (TLRs), receptor for advanced glycation end products (RAGE), P2X purinoceptor 7 (P2X7), the NLR family pyrin domain-containing 3 (NLRP3) inflammasome, and the cyclic GMP–AMP synthase (cGAS)–stimulator of interferon genes (STING) pathway (10). Downstream signaling converges on nuclear factor kappa B (NF-κB), mitogen-activated protein kinase (MAPK) pathways, inflammasome activation, type I interferon responses, and cytokine production, thereby promoting KC activation, neutrophil recruitment, neutrophil extracellular trap (NET)-mediated amplification, endothelial injury, thromboinflammation, and impaired tissue repair (11, 12).

However, DAMPs should not be viewed solely as upstream inflammatory triggers. Their biological and clinical significance depends strongly on cellular source, molecular form, release timing, tissue compartment, and sampling accessibility (11). HMGB1 may be actively secreted by stressed immune or parenchymal cells or passively released from necrotic hepatocytes, and its inflammatory activity is shaped by its redox state. mtDNA reflects mitochondrial disruption and may appear rapidly after reperfusion. In contrast extracellular histones, nucleosomes, and NET-derived DNA may indicate chromatin injury, neutrophil-driven amplification, and endothelial toxicity (8, 12). Extracellular matrix-derived DAMPs may emerge later, reflecting sinusoidal injury, biliary vulnerability, matrix remodeling, and fibrotic repair responses. Thus, different DAMPs are not interchangeable markers of generic tissue injury; instead, they may represent stage-specific molecular signatures of distinct pathological events across cold ischemia, warm ischemia, early reperfusion, delayed inflammation, and repair or remodeling phases (7, 13).

This spatiotemporal perspective is particularly relevant in the current era of dynamic organ preservation and graft assessment. Normothermic machine perfusion (NMP) and hypothermic oxygenated machine perfusion (HOPE) have shifted liver preservation from passive cold storage toward active metabolic support, viability testing, and therapeutic intervention (14). These platforms provide opportunities to quantify perfusate-accessible DAMPs before implantation, monitor mitochondrial and endothelial injury in real time, assess the quality of marginal grafts, and potentially remove or neutralize injurious mediators before systemic reperfusion (15). In this context, circulating and perfusate-derived DAMPs may serve not only as indicators of established injury but also as dynamic molecular readouts that predict EAD, post-reperfusion syndrome, ischemic biliary complications, and the need for targeted perioperative interventions (16, 17).

Recent reviews, including one by Huang et al. and our previous NET-focused review, have summarized sterile inflammation, regulated cell-death pathways, NET biology, and broad therapeutic strategies in liver ischemia–reperfusion injury (18, 19). The present review adopts a distinct, DAMP-centered, and more translationally oriented perspective. Rather than providing another general overview of sterile inflammation, modes of cell death, or NET biology, we focus on DAMPs as measurable, stage-specific, and therapeutically actionable signatures of hepatic injury. Specifically, we organize DAMP biology according to molecular source, release timing, sensing pathway, sampling compartment, biomarker utility, and intervention window. This framework allows DAMPs to be interpreted not merely as inflammatory mediators, but as molecular interfaces linking ischemic stress, reperfusion injury, graft-quality assessment, machine-perfusion monitoring, early complication prediction, and precision intervention.

Accordingly, this review first outlines a conceptual framework for understanding DAMPs as spatiotemporal injury signatures in HIRI. We then summarize major DAMP categories and their release patterns across ischemia, reperfusion, delayed inflammation, and repair or fibrosis phases. Next, we discuss how DAMPs drive cellular and vascular amplification through KCs, LSECs, neutrophils, platelets, cholangiocytes, and hepatic stellate cells, followed by a focused analysis of DAMP-sensing pathways and therapeutic windows. Finally, we evaluate circulating and perfusate-accessible DAMPs as biomarkers, review DAMP-targeted therapeutic strategies, and highlight key requirements for clinical implementation, including assay standardization, well-defined sampling windows, multicenter validation, and rational integration with machine perfusion.

## 2 Conceptual framework: DAMPs as spatiotemporal injury signatures in HIRI

### 2.1 Definition and translational significance of DAMPs

DAMPs are endogenous molecules that acquire immunological significance upon release from injured cells, exposure on altered membranes, fragmentation from the extracellular matrix, or chemical modification during tissue stress (20). Under physiological conditions, many DAMPs are compartmentalized within the nucleus, mitochondria, cytoplasm, secretory granules, or extracellular matrix, where they support structural integrity, metabolic activity, or tissue homeostasis (21). During HIRI, ischemia-induced metabolic failure and reperfusion-induced oxidative stress disrupt this compartmentalization, allowing intracellular or matrix-restricted molecules to emerge as extracellular or cytosolic danger signals (22).

In the liver, DAMPs provide critical insights because HIRI is not merely a consequence of hepatocyte death (23). Rather, it is driven by dynamic communication among hepatocytes, LSECs, KCs, recruited macrophages, neutrophils, platelets, cholangiocytes, and hepatic stellate cells (24). DAMPs function as a molecular language of this communication, linking hepatocellular stress to macrophage activation, mitochondrial disruption to nucleic acid sensing, extracellular matrix injury to leukocyte recruitment, and neutrophil activation to thromboinflammation (25).

From a translational perspective, DAMPs have three interrelated meanings. First, they act as inflammatory mediators by activating PRRs and intracellular sensors (6). Second, they serve as injury readouts because their appearance in blood, bile, graft perfusate, or tissue reflects specific forms of cellular or matrix damage (14). Third, they offer therapeutic opportunities because their release, persistence, receptor engagement, and downstream signaling can be targeted. Thus, DAMPs should be interpreted as stage-specific molecular signatures rather than generic inflammatory molecules (7, 13).

### 2.2 Major DAMP classes in HIRI

DAMPs in HIRI can be grouped according to their dominant source and biological meaning. Although these categories overlap *in vivo*, they help clarify how different injury compartments contribute to sterile inflammation and graft dysfunction.

The first category comprises nuclear and nucleic acid-associated DAMPs, including HMGB1, extracellular histones, nucleosomes, cfDNA, and chromatin-derived components (7). These molecules reflect disruption of nuclear integrity, chromatin organization, membrane stability, or NET formation. HMGB1 is particularly important because it can be actively secreted by stressed cells or passively released from necrotic cells, and its inflammatory activity is strongly influenced by its molecular form and redox status (20).

The second category comprises mitochondrial and metabolic DAMPs, including mtDNA, ATP, oxidized mitochondrial components, ROS-derived products, and lipid peroxidation-derived mediators (26). These signals are especially relevant during early reperfusion, when mitochondrial permeability transition, calcium overload, oxidative stress, and metabolic reactivation converge (27). They indicate mitochondrial injury and also amplify inflammation through inflammasome activation, nucleic acid sensing, and propagation of oxidative damage (28, 29).

The third category includes extracellular matrix-derived and protein-associated DAMPs, such as S100A8/A9, heat shock proteins, hyaluronan fragments, fibronectin extra domain A-containing fragments, laminin fragments, fibrinogen-derived products, and other matrix-remodeling molecules (30). These DAMPs reflect injury to the hepatic microenvironment, including sinusoidal endothelial damage, leukocyte adhesion, platelet activation, vulnerability of the biliary system, hepatic stellate cell activation, and late repair or fibrosis-associated remodeling. In severe reperfusion injury, DAMPs may also occur as multiprotein or nucleic acid–protein complexes, especially NET-associated DNA, histones, myeloperoxidase, neutrophil elastase, and S100 proteins. In this review, NETs are primarily viewed as DAMP-bearing and DAMP-amplifying structures within a broader DAMP-centered framework (30, 31).

### 2.3 Spatiotemporal release across ischemia, reperfusion, and repair

The meaning of a DAMP depends not only on its molecular identity but also on when and where it appears (7). HIRI evolves through overlapping phases, including cold ischemia, warm ischemia, early reperfusion, delayed inflammatory propagation, and repair or remodeling. Each phase is associated with a distinct DAMP profile (13–16).

During cold ischemia, metabolic suppression, oxygen deprivation, and preservation-related stress prime the graft for subsequent injury (4). ATP depletion, mitochondrial destabilization, early HMGB1 translocation, low-level cfDNA release, and endothelial-derived danger cues can initiate before implantation (5). During warm ischemia, especially in DCD grafts or vascular clamping during hepatic surgery, hepatocytes and LSECs are increasingly vulnerable to mitochondrial permeability transition, calcium overload, and redox imbalance (7, 9).

Early reperfusion represents the critical window in which metabolic injury is rapidly converted into sterile inflammation. Oxygen reintroduction triggers ROS production, mitochondrial injury, calcium dysregulation, lipid peroxidation, and membrane damage, resulting in the release of mtDNA, ATP, oxidized mitochondrial components, and lipid-derived DAMPs (4, 5). These signals activate KCs, inflammasome pathways, endothelial cells, and recruited innate immune cells (26–29).

During delayed reperfusion, persistent hepatocyte death, neutrophil recruitment, NET formation, endothelial disruption, and microvascular thrombosis lead to increased extracellular histones, nucleosomes, cfDNA, S100A8/A9, proteases, and other inflammatory protein DAMPs (11). In the repair and remodeling phase, extracellular matrix fragments, heat shock proteins, fibronectin-derived molecules, hyaluronan fragments, and persistent S100-family proteins may become more prominent (12). These late signals may support debris clearance and tissue repair; however, excessive or poorly cleared DAMPs can sustain inflammation, biliary injury, graft dysfunction, and fibrotic remodeling (30, 31).

### 2.4 Sampling compartments and clinical accessibility

A major translational advantage of DAMPs is their measurability in clinically accessible compartments. Peripheral blood, including serum and plasma, is the most practical compartment for postoperative monitoring (7). Circulating HMGB1, mtDNA, cfDNA, histones, nucleosomes, S100A8/A9, and inflammatory protein complexes can indicate hepatocellular injury, mitochondrial damage, endothelial toxicity, neutrophil-driven amplification, and systemic inflammatory burden (12). However, blood-based measurements are influenced by dilution, clearance kinetics, hemolysis, transfusion, infection, renal function, systemic inflammation, and operative variability (30, 31).

Graft perfusate has become increasingly important in the era of machine perfusion. During NMP or HOPE, repeated perfusate sampling can detect graft-derived DAMP release before implantation and before conventional postoperative tests are informative (13). Perfusate DAMPs may help assess mitochondrial integrity, hepatocellular necrosis, endothelial injury, inflammatory potential, and the need for prolonged perfusion or pharmacological conditioning (17).

Bile may provide complementary information on cholangiocyte injury and ischemic biliary complications because biliary vulnerability is not always reflected by serum aminotransferase levels (13). Tissue-based assessment, including biopsy, immunostaining, spatial transcriptomics, proteomics, and imaging, remains essential for defining DAMP sources and their spatial relationship to necrosis, immune infiltration, platelet deposition, sinusoidal injury, and extracellular matrix remodeling. Together, these compartments enable time- and context-specific DAMP interpretation, moving beyond universal single-marker use (15, 16).

### 2.5 Working model of this review

This review adopts a source–time–sensor–sample–intervention model to organize DAMP biology in HIRI. The source dimension identifies which cell or tissue compartment generates a specific DAMP. Hepatocytes mainly release nuclear, mitochondrial, and metabolic DAMPs; LSECs contribute vascular and matrix-associated DAMPs; neutrophils contribute NET-associated DAMP complexes; and cholangiocytes and hepatic stellate cells contribute to biliary and remodeling-associated DAMP responses.

The time dimension differentiates DAMPs initiating injury from those associated with amplification or repair. DAMPs appearing during cold preservation may indicate graft vulnerability; those released during early reperfusion may reflect mitochondrial rupture and acute metabolic injury; and later DAMPs may indicate neutrophil amplification, endothelial damage, biliary stress, or matrix remodeling.

The sensor dimension links DAMP classes to PRRs and intracellular sensing systems, including TLRs, RAGE, P2X7, the NLRP3 inflammasome, cGAS–STING signaling, integrin-dependent sensing, and coagulation-associated pathways. These pathways represent sensing modules embedded within specific cellular and temporal contexts.

**Figure 1:**
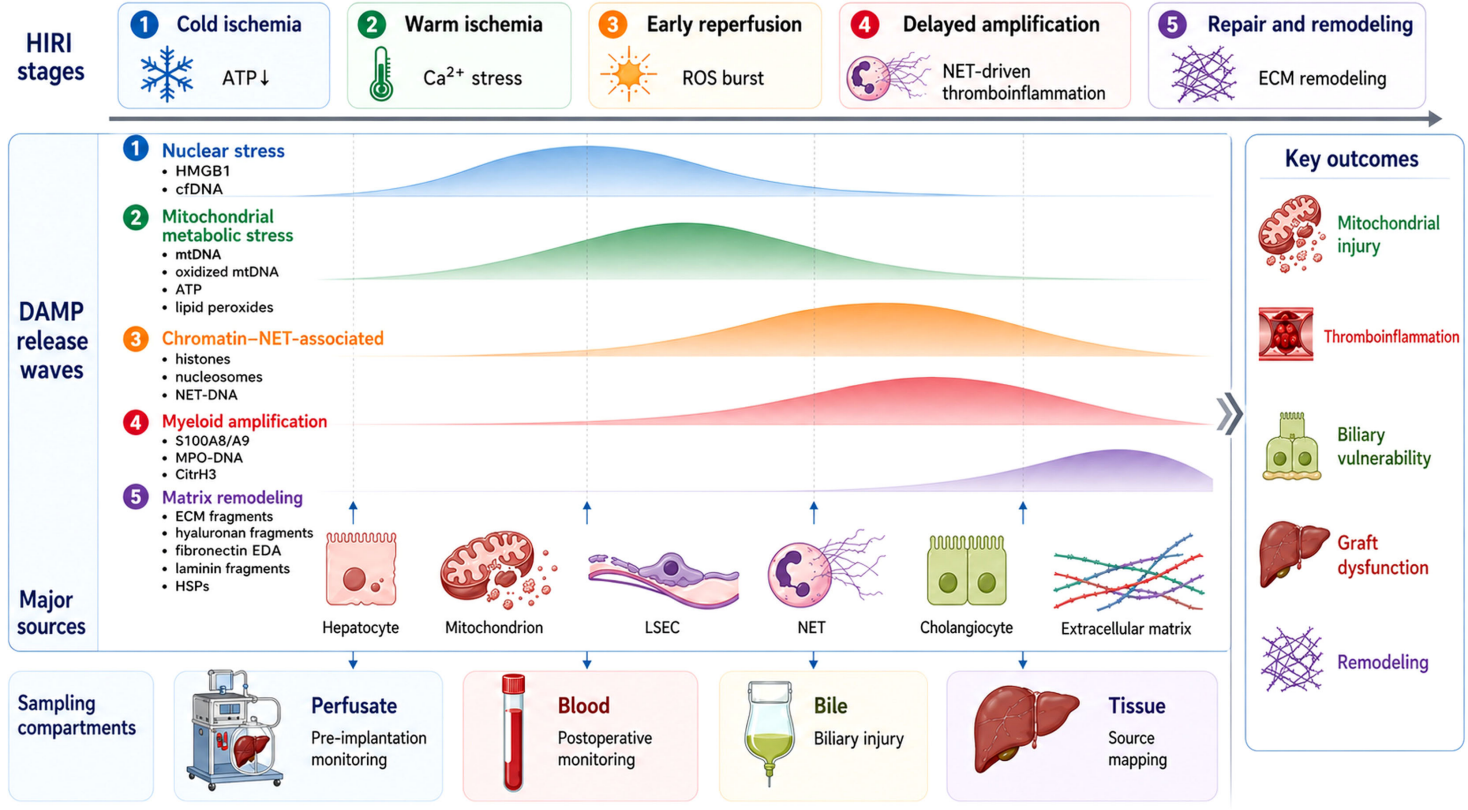
Spatiotemporal release map of damage-associated molecular patterns during hepatic ischemia–reperfusion injury. Damage-associated molecular pattern (DAMP) waves emerge throughout cold ischemia, warm ischemia, early reperfusion, delayed inflammatory amplification, and repair/remodeling during hepatic ischemia–reperfusion injury (HIRI). Early nuclear stress is characterized by high-mobility group box 1 (HMGB1) and cell-free DNA (cfDNA), whereas early reperfusion is dominated by mitochondrial and metabolic signals, including mitochondrial DNA (mtDNA), oxidized mtDNA, adenosine triphosphate (ATP), and lipid peroxidation products. Delayed inflammatory amplification is marked by DAMPs associated with chromatin and neutrophil extracellular trap (NET)-associated DAMPs, including histones, nucleosomes, NET-DNA, S100A8/A9, myeloperoxidase-DNA (MPO-DNA), and citrullinated histone H3 (CitH3). During repair and remodeling, extracellular matrix (ECM) fragments, hyaluronan fragments, fibronectin extra domain A (EDA), laminin fragments, and heat shock proteins (HSPs) become more prominent. These stage-specific DAMP signatures arise from hepatocytes, mitochondria, liver sinusoidal endothelial cells (LSECs), NETs, cholangiocytes, and ECM compartments, and may be detected in perfusate, blood, bile, and tissue to facilitate mechanistic interpretation of mitochondrial injury, thromboinflammation, biliary vulnerability, graft dysfunction, and remodeling.

The sample dimension addresses where DAMPs can be measured—blood, perfusate, bile, or tissue—and whether that compartment captures the relevant injury process. The intervention dimension addresses how DAMP biology can be modified by limiting release, stabilizing mitochondria, reducing oxidative stress, neutralizing extracellular DAMPs, enhancing clearance, blocking dominant sensing pathways, protecting endothelial or biliary compartments, or integrating DAMP-directed therapy with machine perfusion. This model provides the conceptual basis for interpreting DAMPs as dynamic molecular signatures that connect injury stage, biomarker development, and therapeutic opportunity in HIRI (**Figure 1**).

## 3 Major DAMP categories and release patterns in hepatic IRI

### 3.1 Nuclear and nucleic-acid DAMPs: early indicators of hepatocellular and chromatin injury

Nuclear and nucleic acid-associated DAMPs are among the earliest molecular signals released during HIRI. Their appearance reflects disruption of nuclear integrity, chromatin organization, membrane stability, and release of chromatin associated with neutrophil extracellular traps (NETs) (7). Unlike conventional liver enzymes, which mainly indicate hepatocellular leakage, these DAMPs may provide information about injury source, timing, molecular form, and vascular inflammatory consequences (20–22).

HMGB1 is the prototypical nuclear DAMP in HIRI. Under physiological conditions, HMGB1 is mainly localized in the nucleus, where it participates in chromatin organization, transcriptional regulation, and DNA repair (32). During ischemic stress, oxidative injury, necrosis, or immune-cell activation, HMGB1 can translocate to the cytoplasm and subsequently be released via active secretion or passive leakage (20). This dual release mode is biologically important: active secretion may indicate stress-programmed inflammatory activation, whereas passive release directly reflects membrane disruption and necrotic injury. Its inflammatory activity also depends on redox state and post-translational modification; therefore, total HMGB1 levels alone may be less informative than kinetic or isoform-sensitive assessment, especially in transplantation, where injury progresses through preservation, implantation, and reperfusion (33).

CfDNA provides another important nuclear injury signal. Nuclear cfDNA may arise from necrosis, apoptosis-associated fragmentation, endothelial injury, and NET release. In liver transplantation, donor-derived cfDNA is particularly attractive because it can provide a graft-specific injury readout that is less confounded by recipient systemic inflammation (34). However, cfDNA interpretation requires attention to fragment size, tissue origin, sampling time, ischemia duration, perioperative transfusion, renal clearance, infection, and concurrent tissue injury. Thus, cfDNA should not be viewed only as a nonspecific cell-death marker; when interpreted dynamically, it may help distinguish graft injury, immune activation, and delayed inflammatory propagation (35).

Extracellular histones and nucleosomes represent a more cytotoxic chromatin-derived DAMP subset. Histones are essential for chromatin packaging intracellularly, but once released extracellularly, they can damage hepatocytes, liver sinusoidal endothelial cells (LSECs), and vascular structures through charge-dependent membrane interactions, coagulation activation, endothelial toxicity, and inflammatory receptor engagement (24). In HIRI, histones may originate from necrotic hepatocytes, injured endothelial cells, or NETs. Their appearance is therefore closely linked to thromboinflammation, sinusoidal obstruction, and microcirculatory dysfunction (32). Overall, nuclear and nucleic acid-associated DAMPs are most useful when interpreted according to their temporal profile: HMGB1 may increase during ischemic stress and early reperfusion, whereas cfDNA, nucleosomes, histones, and NET-associated chromatin may become more prominent during delayed inflammatory and vascular amplification (36).

### 3.2 Mitochondrial and metabolic DAMPs: reperfusion-sensitive amplifiers

Mitochondrial and metabolic DAMPs are central to the transition from ischemic stress to reperfusion-associated inflammatory amplification (9). The liver is a highly metabolic organ, and hepatocytes depend heavily on mitochondrial oxidative phosphorylation, lipid metabolism, bile acid homeostasis, and detoxification. During ischemia, mitochondrial respiration declines, ATP production falls, ionic gradients collapse, and antioxidant capacity is impaired (10). Reperfusion then abruptly restores oxygen supply, but this restoration may trigger ROS generation, calcium overload, mitochondrial permeability transition, lipid peroxidation, and release of mitochondrial danger signals (37).

MtDNA is one of the most informative mitochondrial DAMPs in HIRI. Because mitochondria possess bacterial-like molecular features, including unmethylated cytosine-phosphate-guanine motifs, mtDNA is recognized as an immunostimulatory signal when it enters the cytosol, extracellular space, blood, bile, or graft perfusate. Reperfusion-induced mitochondrial rupture, permeability transition pore opening, defective mitophagy, and vesicular release may all contribute to mtDNA exposure (37). Oxidized mtDNA may be particularly relevant because it reflects both mitochondrial disruption and reperfusion-associated oxidative stress. In the transplant setting, mtDNA is attractive because it can rise early and be measurable in circulating blood, preservation fluid, perfusate, or bile, thereby linking mitochondrial vulnerability to graft assessment (37, 38).

Extracellular ATP is another key metabolic DAMP. Intracellularly, ATP is an energy carrier; extracellularly, it functions as a danger signal, released via membrane damage, pannexin channels, vesicles, or necrotic leakage (25). In the reperfused liver, extracellular ATP can activate P2X7, promote potassium efflux, and facilitate NLRP3 inflammasome activation. Although ATP is difficult to use as a stable biomarker because it is rapidly degraded by ectonucleotidases, it holds substantial pathway-level significance because it links metabolic failure, inflammasome activation, and secondary DAMP release (39).

ROS and oxidatively modified metabolites further expand the metabolic DAMP landscape. ROS are not classical DAMPs in the strict sense, but they generate and modify DAMPs by oxidizing mtDNA, injuring mitochondrial membranes, altering protein structure, and promoting lipid peroxidation (4). Lipid peroxidation-derived mediators, including aldehyde products and oxidized arachidonic acid metabolites, can aggravate membrane damage and ferroptotic susceptibility. The arachidonate 12-lipoxygenase (ALOX12)–12-hydroxyeicosatetraenoic acid (12-HETE) axis is relevant because it links calcium-dependent enzymatic activity, lipid peroxidation, and hepatocyte injury during reperfusion. In steatotic, aged, or donation-after-circulatory-death grafts, impaired mitochondrial reserve and abundant lipid substrates may amplify this process (5). Therefore, mitochondrial and metabolic DAMPs should be interpreted as dynamic indicators of metabolic reserve, reperfusion sensitivity, and graft resilience rather than as generic cell-death products (4, 5).

### 3.3 Extracellular matrix-derived and protein DAMPs: microenvironmental remodeling signals

Extracellular matrix (ECM)-derived and protein DAMPs reflect injury to the hepatic microenvironment, not merely intracellular damage (40). Their release indicates disruption of sinusoidal endothelial integrity, basement membrane exposure, leukocyte adhesion, platelet activation, biliary vulnerability, coagulation activation, and stromal remodeling (41, 42). This category is important because clinically relevant HIRI is not limited to hepatocyte necrosis; it also includes endothelial swelling, sinusoidal congestion, cholangiocyte injury, platelet-neutrophil interactions, and hepatic stellate cell activation (43, 44).

S100A8/A9 is a prominent protein DAMP associated with myeloid activation. It can be released by activated neutrophils, monocytes, macrophages, and stressed parenchymal or biliary cells (45). Through Toll-like receptor 4 (TLR4) and receptor for advanced glycation end-products (RAGE) signaling, S100A8/A9 amplifies chemokine production, macrophage activation, neutrophil recruitment, and sustained inflammatory propagation (46). In HIRI, it is best interpreted as a marker and mediator of myeloid amplification rather than a simple indicator of hepatocyte death. Its persistence after early reperfusion may suggest unresolved innate immune activation and the risk of ongoing endothelial or biliary injury (43).

Heat shock proteins are another stress-related DAMP class. Intracellularly, they act as molecular chaperones that support protein folding and cytoprotection. Extracellularly, they may modulate antigen-presenting cells, scavenger receptors, and innate immune pathways (27). Their role is context-dependent: controlled heat shock responses may support cell survival and repair, whereas excessive extracellular release may reinforce inflammatory activation. Therefore, heat shock proteins require phase-specific interpretation (44).

Matrix-derived DAMPs, including hyaluronan fragments, fibronectin extra domain A-containing fragments, laminin fragments, collagen-derived peptides, proteoglycan fragments, fibrinogen-derived products, and D-dimer-like fragments, arise when ischemia, oxidative stress, protease activity, and inflammatory infiltration remodel the hepatic matrix (45). These fragments can interact with integrins, TLRs, RAGE, and coagulation-related receptors. In the hepatic sinusoid, ECM fragmentation may enhance leukocyte adhesion, platelet deposition, endothelial barrier dysfunction, and microvascular obstruction. In the biliary compartment, matrix damage may contribute to cholangiocyte stress and ischemic biliary complications (46). Compared with early mitochondrial signals, many ECM-derived DAMPs are more informative during delayed inflammation, endothelial injury, biliary stress, and repair or fibrosis. Their persistence may help distinguish transient biochemical injury from progressive graft dysfunction, ischemic cholangiopathy, or fibrotic remodeling (47).

### 3.4 Integrated DAMP signatures rather than single molecules

HIRI is unlikely to be captured accurately by a single DAMP. The ischemic and reperfused liver releases multiple DAMPs simultaneously, and their relative abundance changes across time, compartment, and injury severity (48). A high HMGB1 level may indicate hepatocellular stress or necrosis, but it cannot pinpoint whether mitochondrial rupture, endothelial injury, neutrophil activation, biliary damage, or matrix remodeling is dominant (7–12). Similarly, mtDNA reflects mitochondrial disruption but does not fully represent chromatin toxicity, thromboinflammation, or late stromal injury. A multimarker approach is therefore more consistent with the biology of HIRI (46).

Integrated DAMP signatures allow for phase-specific interpretation. An early hepatocellular stress signature may include HMGB1 translocation, low-level cfDNA release, ATP-related stress signals, and early mitochondrial injury markers (41). An early reperfusion signature may be enriched for mtDNA, oxidized mtDNA, extracellular ATP-related pathway activation, ROS-modified metabolites, and lipid peroxidation products (11). A delayed inflammatory amplification signature may include extracellular histones, nucleosomes, NET-associated DNA, S100A8/A9, myeloperoxidase-DNA complexes, and inflammatory protein DAMPs. A repair or remodeling signature may include ECM fragments, heat shock proteins, persistent S100-family proteins, and markers of endothelial or biliary stress (12, 30).

This phase-based interpretation has practical implications. In blood, combined DAMP panels may help distinguish transient biochemical injury from evolving early allograft dysfunction, post-reperfusion syndrome, ischemic biliary complications, or persistent inflammation (34). In graft perfusate, DAMP combinations may support viability assessment before implantation, especially when paired with lactate clearance, bile production, vascular resistance, oxygen consumption, perfusate transaminases, and histology (35). In bile, DAMPs may capture cholangiocyte vulnerability not fully reflected by serum aminotransferases. In tissue, spatial DAMP patterns may reveal which type of injury—hepatocellular, endothelial, immune, biliary, or stromal—is dominant (16).

DAMPs are not uniformly pathogenic. Transient DAMP release may support debris clearance, immune resolution, matrix remodeling, and repair, whereas excessive or persistent DAMP signaling promotes maladaptive amplification (28). The key translational question is not whether a DAMP is present, but whether its molecular form, concentration, location, and timing indicate adaptive repair or progressive injury. This integrated view provides the foundation for interpreting DAMPs as coordinated spatiotemporal signatures, connecting molecular injury to biomarker development and clinical translation in HIRI (31). The major DAMP signatures, dominant release windows, accessible samples, and translational meanings are summarized in **Table 1**.

**Table 1:** Major damage-associated molecular pattern (DAMP) signatures in hepatic ischemia–reperfusion injury.

DAMP signature	Dominant source	Main release window	Accessible sample	Translational meaning	References
HMGB1	Hepatocytes, macrophages	Cold ischemia to early reperfusion	Blood, perfusate, tissue	Early hepatocellular stress; inflammatory priming	(32, 33)
cfDNA and donor-derived cfDNA	Injured hepatocytes, graft tissue	Early reperfusion to delayed injury	Blood, perfusate	Graft injury burden; dynamic injury monitoring	(34, 35)
mtDNA and oxidized mtDNA	Damaged mitochondria	Early reperfusion	Blood, perfusate, bile	Mitochondrial rupture; reperfusion sensitivity	(9, 10)
ATP	Injured hepatocytes, stressed cells	Early reperfusion	Perfusate, tissue	Metabolic danger; inflammasome activation	(25, 39)
Histones and nucleosomes	Necrotic cells, NETs	Delayed amplification	Blood, tissue	Chromatin toxicity; endothelial injury	(24, 36)
NET-DNA, MPO-DNA, CitH3	Neutrophils, NETs	Delayed amplification	Blood, tissue	NET burden; thromboinflammation	(11, 12)
S100A8/A9	Neutrophils, monocytes, macrophages	Delayed inflammation	Blood, tissue	Myeloid amplification; persistent sterile inflammation	(30, 31)
ECM fragments	Matrix, LSECs, stromal cells	Repair and remodeling	Perfusate, bile, tissue	Matrix injury; biliary stress; remodeling risk	(45, 46, 49)
HSPs	Stressed hepatocytes, immune cells	Delayed injury to repair	Blood, tissue	Stress response; inflammation or repair context	(44, 50)
Lipid peroxidation products	Injured hepatocytes, mitochondria	Early reperfusion	Blood, perfusate, tissue	Ferroptotic injury; metabolic graft vulnerability	(4, 5)

ATP, adenosine triphosphate; cfDNA, cell-free DNA; CitH3, citrullinated histone H3; DAMP, damage-associated molecular pattern; ECM, extracellular matrix; EDA, extra domain A; HMGB1, high-mobility group box 1; HSPs, heat shock proteins; MPO-DNA, myeloperoxidase-DNA; mtDNA, mitochondrial DNA; NET, neutrophil extracellular trap.

## 4 DAMP-driven cellular and vascular amplification modules

### 4.1 Kupffer cells and monocyte-derived macrophages: primary DAMP-processing hubs

KCs and recruited monocyte-derived macrophages are key cellular hubs that translate DAMP release into inflammatory amplification during HIRI (51). Their sinusoidal location enables immediate exposure to hepatocyte-, mitochondrial-, endothelial-, and matrix-derived danger signals after reperfusion (52). Rather than responding to a single ligand, macrophages integrate multiple DAMP inputs, leading to cytokine production, chemokine gradients, oxidative stress, inflammasome activation, and the recruitment of additional innate immune cells (53).

Recent studies in human liver transplantation support this concept at the molecular level (54). Disulfide HMGB1, rather than total HMGB1 alone, correlates with the severity of ischemia–reperfusion injury and can induce TLR4-dependent tumor necrosis factor-α (TNF-α) production by macrophages (8). Further work has shown that disulfide HMGB1 signals through both TLR4 and TLR9, generating inflammatory macrophages capable of bridging innate and adaptive immune responses (8, 55–57). These findings are important because they define macrophage activation as a DAMP-form-specific process rather than merely as a response to nonspecific necrotic debris (58, 59).

Macrophage activation is also regulated by upstream hepatocyte stress programs, including metabolic stress, endoplasmic reticulum stress, and DAMP release, which together shape macrophage recruitment and inflammatory output (51, 56). This finding links metabolic reprogramming, DAMP modification, and macrophage recruitment within a single source–sensor axis (51, 60).

Functionally, activated KCs and monocyte-derived macrophages release TNF-α, interleukin-1β (IL-1β), interleukin-6 (IL-6), chemokines, ROS, and inflammasome-related mediators (46). These outputs promote neutrophil recruitment, LSEC activation, platelet adhesion, and secondary hepatocyte injury (51). However, macrophages are not uniformly pathogenic. During the later phase of injury, they contribute to efferocytosis, DAMP clearance, inflammatory resolution, and repair. Thus, macrophages should be viewed as dynamic DAMP-processing hubs: early after reperfusion, they amplify sterile inflammation; later, they may support resolution or fibrosis, depending on the persistence of danger signaling (55, 56).

### 4.2 Hepatocytes and LSECs: the parenchymal–vascular injury interface

Hepatocytes and LSECs form the parenchymal–vascular interface that determines whether DAMP release remains localized or progresses into clinically significant graft dysfunction (57). Hepatocytes are the dominant source of nuclear, mitochondrial, and metabolic DAMPs, whereas LSECs are sensitive targets and amplifiers of DAMP-induced microvascular injury (46). This bidirectional relationship explains why HIRI severity is determined not by hepatocyte necrosis alone but also by sinusoidal perfusion, endothelial integrity, leukocyte adhesion, platelet activation, and tissue oxygen delivery (60).

During ischemia, hepatocytes undergo ATP depletion, mitochondrial depolarization, ionic imbalance, endoplasmic reticulum stress, and impaired antioxidant capacity (57). Upon reperfusion, oxygen reintroduction triggers ROS production, mitochondrial permeability transition, calcium overload, and lipid peroxidation (19). A recent mechanistic study identified transient receptor potential melastatin 2 (TRPM2)-mediated calcium influx as a driver of HIRI, acting via ALOX12-dependent mitochondrial lipid peroxidation and ferroptotic injury. This supports the view that hepatocyte-derived DAMP release is shaped by defined metabolic, ionic, and lipid-peroxidation programs rather than passive necrosis alone (5).

LSECs are uniquely vulnerable during reperfusion because they regulate sinusoidal tone, filtration, scavenger function, leukocyte trafficking, and antithrombotic balance. Endothelial swelling, reduced nitric oxide (NO) bioavailability, glycocalyx injury, adhesion molecule expression, and von Willebrand factor (vWF) release convert molecular danger signals into sinusoidal congestion and heterogeneous tissue perfusion (52). Experimental evidence demonstrating that endothelial senescence reversal, endothelial Notch modulation, and VEGF-C-mediated vascular protection can alter HIRI severity supports endothelial preservation as a strategy to reduce downstream parenchymal injury (57–59).

From a DAMP perspective, LSECs have three linked roles: they sense local danger signals, present or release vascular and matrix-associated DAMPs after injury, and translate DAMP exposure into leukocyte adhesion, platelet binding, permeability changes, and microvascular obstruction (57). This interface is particularly relevant in steatotic, aged, or DCD grafts, where impaired mitochondrial reserve and endothelial vulnerability can magnify the impact of the DAMP burden (60, 61).

### 4.3 Neutrophils and NETs: DAMP-amplifying role, not an independent focus

Neutrophils are rapidly recruited during reperfusion, driven by macrophage-derived cytokines, DAMP-induced chemokines, complement activation, endothelial adhesion molecules, platelet interactions, and mitochondrial danger signals (62). Once retained in sinusoids or infiltrating the parenchyma, they amplify injury through ROS generation, degranulation, protease release, endothelial adhesion, and NET formation (63).

In this review, NETs are considered primarily as structures that bear and amplify DAMPs, rather than an independent mechanistic focus. NETs contain extracellular DNA, histones, myeloperoxidase (MPO), neutrophil elastase, S100 proteins, and other nuclear or granular components (62). These components injure hepatocytes and LSECs, activate macrophages, promote platelet adhesion, enhance coagulation, and sustain inflammatory signaling. Studies have shown that DAMPs can induce NET formation in liver ischemia–reperfusion injury and that peptidylarginine deiminase 4 (PAD4) inhibition or deoxyribonuclease I (DNase I) reduces HMGB1- and histone-mediated liver injury (64).

NET-associated DAMPs reinforce several pathogenic loops. Extracellular histones disrupt membranes and endothelial barriers; NET-derived DNA and histones promote platelet adhesion and microthrombus formation; MPO and neutrophil elastase aggravate oxidative and proteolytic injury; and NET-derived nucleic acids can activate DNA-sensing pathways (65). Nevertheless, neutrophils are not uniformly harmful. Limited early neutrophil responses may support antimicrobial defense, debris containment, and tissue stabilization (66). Therefore, the translational challenge is to identify when neutrophil-derived DAMPs shift from protective containment to maladaptive amplification. Time-resolved markers such as MPO–DNA complexes, CitH3, extracellular DNA, and histone-associated signals may be useful when considered alongside mitochondrial and endothelial DAMPs (67).

### 4.4 Platelet–endothelial–coagulation interface: DAMPs and thromboinflammation

The vascular consequences of HIRI are central to graft dysfunction. Reperfusion restores macroscopic inflow, yet sinusoidal perfusion may remain heterogeneous because of endothelial swelling, vasoconstriction, leukocyte adhesion, platelet aggregation, NET deposition, and microthrombus formation. DAMPs offer a molecular explanation for this mismatch between restored blood flow and impaired tissue-level oxygen delivery (68).

Platelets interact closely with LSECs and neutrophils during hepatic reperfusion. NET-dependent platelet activation and microvascular thrombus formation mechanistically link DAMP release to thromboinflammatory injury (65, 67, 68). Nicotinamide adenine dinucleotide phosphate oxidase 2 (NOX2)-dependent neutrophil–platelet interactions further demonstrate how oxidative signaling integrates innate immune activation and thromboinflammatory injury (68, 69).

DAMPs intensify this platelet–endothelial–neutrophil axis through several mechanisms. Extracellular histones and DNA provide charged surfaces that support platelet adhesion and coagulation activation (67). NET structures serve as scaffolds for platelet binding and fibrin deposition. HMGB1 and S100 proteins activate endothelial and immune signaling, increasing chemokine release and adhesion molecule expression. Fibrin degradation products, D-dimer, and complement activation products further connect DAMP biology with coagulation (68, 69). In human liver transplantation, extracellular histones have been clinically associated with primary graft dysfunction, reinforcing their relevance as vascular-toxic DAMPs, not merely inert cell-death products (36).

This module is clinically important because microcirculatory injury contributes to EAD and biliary complications (66). The biliary tree depends on an intact peribiliary vascular plexus; therefore, microvascular obstruction may aggravate cholangiocyte injury even when hepatocellular enzyme levels partially improve. DAMP signatures combining histones, nucleosomes, NET-associated DNA, S100A8/A9, endothelial activation markers, and coagulation-related fragments may therefore provide more information than hepatocyte-derived markers alone (69, 70).

### 4.5 Cholangiocytes, hepatic stellate cells, and late repair or remodeling

Late outcomes after HIRI are frequently determined by biliary injury, incomplete resolution, and stromal remodeling. Cholangiocytes and hepatic stellate cells (HSCs) are therefore important downstream compartments in a DAMP-centered framework (71). They may not be the earliest DAMP sensors, but they influence whether acute injury resolves, progresses to ischemic cholangiopathy, or leaves a profibrotic microenvironment (72).

Cholangiocytes are vulnerable because the biliary epithelium depends on adequate arterial and peribiliary microvascular perfusion. DAMPs may affect cholangiocytes directly through oxidative stress, mitochondrial injury, inflammatory mediators, and extracellular matrix remodeling, or indirectly through endothelial dysfunction and peribiliary vascular compromise (70). Studies measuring mtDNA in perfusate and bile during *ex vivo* NMP suggest that these compartments can reveal graft and biliary injury signals not fully reflected by serum aminotransferase levels (73).

HSCs are traditionally associated with fibrosis, but they also participate in repair and regeneration after ischemia–reperfusion injury. In a murine partial ischemia–reperfusion model, HSC proliferation mediated by Yes-associated protein (YAP) and transcriptional coactivator with PDZ-binding motif (TAZ) contributed to liver repair and regeneration (71). This indicates that HSC activation should not be interpreted solely as harmful fibrosis; its meaning depends on timing, magnitude, and tissue context (72, 73).

Persistent DAMP exposure may shift this reparative program toward maladaptive remodeling. S100-family proteins, extracellular matrix fragments, HMGB1, nucleic acid-containing complexes, and inflammatory cytokines can alter HSC activation, chemokine production, matrix deposition, and crosstalk with macrophages and endothelial cells (28). In fatty liver ischemia–reperfusion injury, targeting the S100A9–TLR2 axis reduced macrophage NLRP3 inflammasome activation and inflammatory injury, supporting the relevance of DAMP–myeloid–stromal crosstalk in metabolically stressed grafts (72, 73).

**Figure 2:**
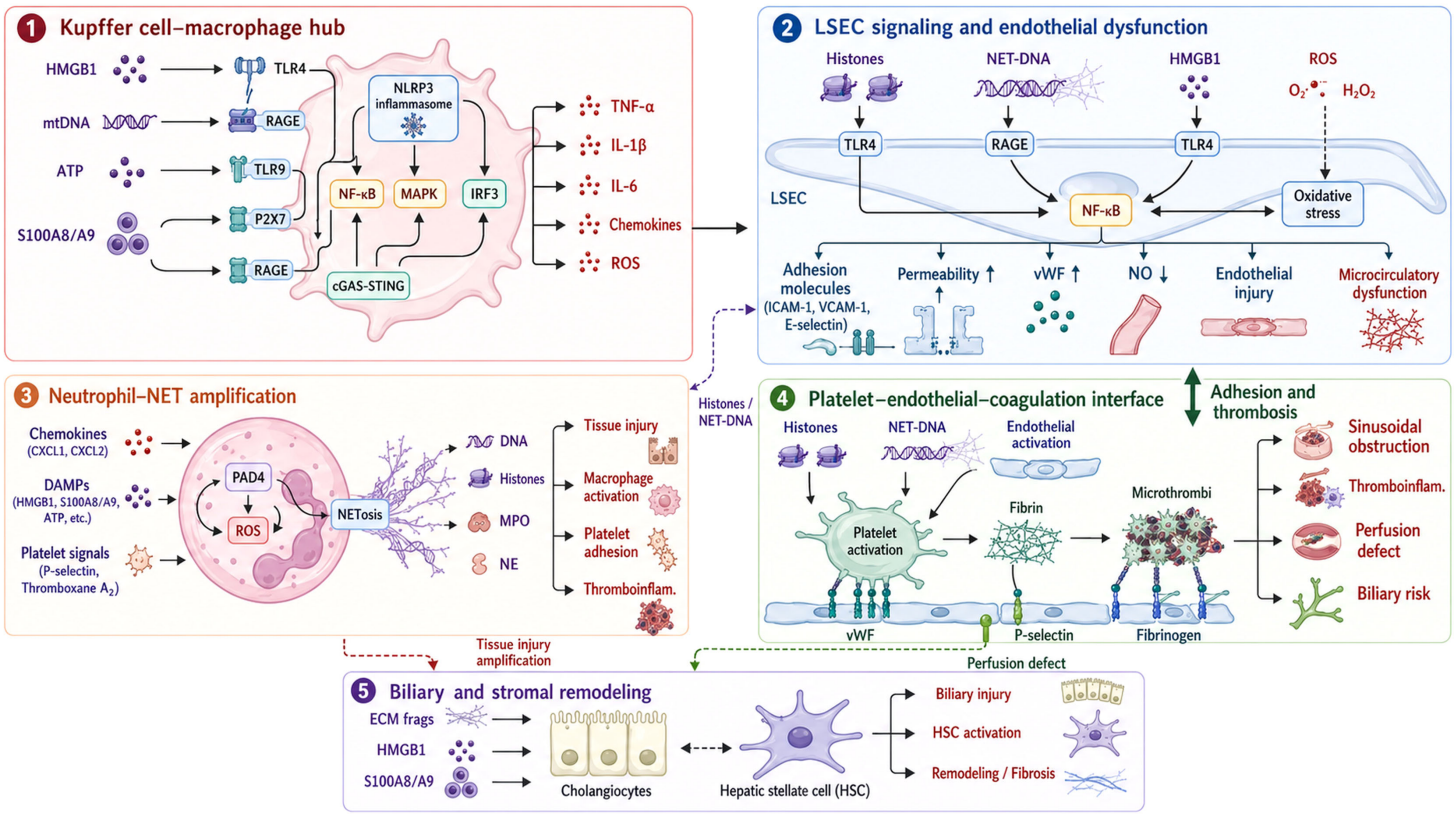
DAMP-triggered signaling networks in key cellular compartments during hepatic ischemia–reperfusion injury. Damage-associated molecular patterns (DAMPs) activate Kupffer cells and macrophages, liver sinusoidal endothelial cells (LSECs), neutrophils, and platelet–endothelial–coagulation interfaces through major sensing pathways, including Toll-like receptor 4 (TLR4), receptor for advanced glycation end products (RAGE), Toll-like receptor 9 (TLR9), P2X purinoceptor 7 (P2X7), cyclic GMP–AMP synthase (cGAS)–stimulator of interferon genes (STING), and NLR family pyrin domain-containing 3 (NLRP3) inflammasome signaling. These interconnected modules drive cytokine production, endothelial dysfunction, neutrophil extracellular trap (NET) formation, thromboinflammation, microcirculatory impairment, biliary injury, and late remodeling. Abbreviations shown in the image include von Willebrand factor (vWF), nitric oxide (NO), reactive oxygen species (ROS), myeloperoxidase (MPO), neutrophil elastase (NE), and peptidylarginine deiminase 4 (PAD4).

Overall, cellular and vascular amplification in HIRI is best understood as an integrated DAMP-processing network (5). Hepatocytes and LSECs initiate and propagate source-specific DAMP release; KCs and recruited macrophages convert DAMP recognition into inflammatory programs; neutrophils and NETs amplify DAMP burden and thromboinflammation; platelets and endothelial cells convert danger exposure into microcirculatory dysfunction; and cholangiocytes and HSCs influence late biliary and remodeling outcomes (36). This modular organization highlights DAMP-sensing pathways as therapeutic windows embedded within specific cellular and temporal contexts (74). These cellular and vascular amplification modules are coordinated through a limited set of dominant DAMP-sensing pathways in Kupffer cells, endothelial cells, neutrophils, and thromboinflammatory compartments, as summarized in **Figure 2**.

## 5 DAMP-sensing pathways and therapeutic windows

### 5.1 HMGB1/S100A8/A9–TLR4/RAGE axis: early inflammatory priming

The HMGB1/S100A8/A9–TLR4/RAGE axis is a major cell-surface sensing module that converts early DAMP release into inflammatory priming during HIRI. This axis is activated mainly by nuclear and protein-based DAMPs derived from injured hepatocytes, activated myeloid cells, neutrophils, endothelial cells, and the remodeled extracellular matrix (75). Its core function is to initiate transcriptional inflammatory programs that promote KC activation, chemokine release, neutrophil recruitment, endothelial activation, and cytokine amplification (76, 77).

HMGB1 is central to this module because its biological activity is determined by its molecular form. Reduced, disulfide and oxidized HMGB1 exert distinct biological effects. In human liver transplantation, disulfide HMGB1 has been associated with histological evidence of HIRI and can induce TLR4-dependent macrophage inflammatory responses (78). Further evidence suggests that disulfide HMGB1 can also signal through TLR9, linking extracellular protein DAMPs to nucleic acid-sensing pathways. These findings support a shift from measuring total HMGB1 toward isoform-specific and pathway-specific interpretation (79). S100A8/A9 adds a second layer of amplification, particularly in neutrophil-rich or steatotic grafts, where persistent myeloid activation may sustain macrophage inflammasome activity and endothelial injury (77, 79).

The therapeutic window for this axis is early and time-sensitive. Inhibition of HMGB1, TLR4, RAGE, or S100A8/A9 signaling may be most rational during preservation, implantation, or early reperfusion, when inflammatory priming is being established (74). Once neutrophil infiltration, thromboinflammation, and matrix remodeling become dominant, blockade of this axis alone is unlikely to be sufficient (80).

### 5.2 ATP–P2X7–NLRP3 axis: metabolic danger and inflammasome amplification

The ATP–P2X7–NLRP3 axis links metabolic collapse to inflammasome activation (81). During ischemia, intracellular ATP depletion reflects bioenergetic failure; after reperfusion, extracellular ATP released from damaged hepatocytes, endothelial cells, or immune cells acts as a danger signal. Through P2X7 activation, extracellular ATP promotes ion flux, potassium efflux, and inflammasome assembly (82).

NLRP3 integrates multiple DAMP-related inputs, including extracellular ATP, mitochondrial ROS, oxidized mtDNA, lysosomal stress, histones, and impaired autophagy or mitophagy (83). In KCs and recruited macrophages, NLRP3 activation drives caspase-1 cleavage, IL-1β maturation, interleukin-18 (IL-18) release, and gasdermin D (GSDMD)-mediated pyroptotic amplification. This process is more than just a downstream inflammatory event; pyroptotic membrane disruption can release additional DAMPs and thereby reinforce sterile inflammation (84).

This module is most actionable during early reperfusion and early inflammatory propagation. Short-term, localized, or graft-directed inhibition may reduce cytokine maturation and secondary DAMP release (85). However, sustained systemic inflammasome blockade could impair host defense, debris clearance, and tissue repair. Therefore, this axis should be interpreted as a transient therapeutic window rather than a target for prolonged global suppression (86, 87).

### 5.3 mtDNA/cfDNA–TLR9/cGAS–STING axis: nucleic acid sensing and interferon responses

mtDNA and cfDNA activate nucleic acid-sensing pathways particularly relevant for understanding the spatiotemporal aspects of HIRI (88). Mitochondrial rupture, oxidative stress, defective mitophagy, and chromatin injury can expose DNA in extracellular or cytosolic compartments. These DNA signals are primarily sensed by TLR9 and the cGAS–STING pathway (89, 90).

TLR9 recognizes unmethylated cytosine–phosphate–guanine (CpG) motifs, which are enriched in mtDNA (91). cGAS detects cytosolic DNA and activates STING, leading to TANK-binding kinase 1 (TBK1) activation, interferon regulatory factor 3 (IRF3) phosphorylation, NF-κB signaling, and type I interferon responses. Experimental studies have shown that blocking mtDNA release or inhibiting cGAS–STING signaling attenuates liver injury, inflammatory activation, and cell death during HIRI (92). This axis may be especially important in aged or grafts susceptible to mitochondrial damage, in which defective mitochondrial quality control increases cytosolic DNA leakage and inflammatory macrophage activation (77, 93).

The therapeutic window for this pathway may begin before implantation if mitochondrial injury is detectable in preservation fluid or machine perfusate. After reperfusion, the first few hours are likely the most critical period, when mitochondrial rupture and DNA leakage are prominent (91). Clinically, mtDNA and cfDNA may therefore serve as both biomarkers of mitochondrial or chromatin injury and guides for modulating DNA-sensing pathways. The major limitation is safety: excessive suppression of DNA sensing may compromise antiviral and antimicrobial defense (93).

### 5.4 Histones, NET-DNA, and endothelial cytotoxicity

Extracellular histones, nucleosomes, and NET-associated DNA form a DAMP module linking chromatin release to endothelial injury and thromboinflammation. Unlike receptor-dominant pathways, this module also causes direct biophysical damage (94). Histones can interact with negatively charged cell membranes, increase permeability, injure hepatocytes and LSECs, activate platelets, and promote coagulation. NET-associated DNA and histones also provide scaffolds for platelet adhesion and fibrin deposition, thereby aggravating sinusoidal obstruction (95).

This module becomes particularly important during delayed reperfusion, when neutrophil recruitment, NET formation, endothelial damage, and microvascular thrombosis amplify the initial injury (46). NET-associated MPO, neutrophil elastase, S100 proteins, and chromatin fragments can sustain oxidative and proteolytic injury while also activating DNA-sensing and inflammasome pathways (94). Thus, chromatin-derived DAMPs elucidate why aminotransferase levels alone may not fully capture clinically relevant microvascular or biliary injury (96).

The therapeutic window for this module is likely later than the early HMGB1 or mtDNA burst and overlaps with neutrophil–endothelial–platelet amplification. Potential approaches include extracellular DNA degradation, histone neutralization, selective inhibition of pathological NET formation, endothelial protection, and antithromboinflammatory strategies (69). Because NETs and coagulation also contribute to antimicrobial defense and hemostasis, interventions should aim to reduce persistent or excessive chromatin burden rather than completely abolish these responses (96).

### 5.5 Convergent outputs and timing-specific pathway selection

Although DAMPs engage distinct receptors, their downstream outputs converge on a limited set of inflammatory programs, including NF-κB activation, MAPK signaling, inflammasome activation, IL-1β and IL-18 maturation, type I interferon production, chemokine induction, endothelial activation, and amplification of cell death. This convergence explains why blockade of a single ligand may be insufficient when multiple DAMPs are released simultaneously (97).

The key implication is that DAMP-sensing pathways should be interpreted according to timing and the dominant injury compartment rather than pathway taxonomy alone. During preservation and pre-reperfusion, the priority is to limit DAMP release by preserving mitochondrial and endothelial integrity (98). During early reperfusion, HMGB1/TLR4/RAGE, mtDNA/cGAS–STING, and ATP/NLRP3 signaling may be most actionable. During delayed reperfusion, histone/NET-DNA-driven endothelial toxicity, platelet activation, and thromboinflammation become more relevant. During repair, broad suppression may be harmful if it interferes with efferocytosis, immune resolution, cholangiocyte recovery, and matrix remodeling (99).

Therefore, the goal is not to identify one universal DAMP pathway for all patients. A more realistic strategy is to match the dominant DAMP signature to the appropriate sampling compartment, injury phase, and intervention window (74). This approach integrates mechanistic DAMP biology with biomarker-guided therapy, machine-perfusion monitoring, and clinical decision-making in liver transplantation and hepatic surgery (100).

## 6 DAMPs as biomarkers for HIRI

### 6.1 Circulating DAMPs for diagnosis and prognosis

Circulating DAMPs provide a minimally invasive window into HIRI. Compared with conventional liver function tests, DAMPs may provide more mechanistic information because they reflect the cellular source, subcellular compartment, and inflammatory context of injury (101). Alanine aminotransferase (ALT), aspartate aminotransferase (AST), bilirubin, lactate, and the international normalized ratio (INR) remain clinically indispensable, but they cannot distinguish mitochondrial rupture from chromatin injury, endothelial toxicity, NET-mediated amplification, or extracellular matrix remodeling. DAMP-based biomarkers may therefore complement, rather than replace, established biochemical and clinical indices (102).

HMGB1 is among the most extensively studied circulating DAMPs in liver transplantation and HIRI (101). Its clinical value depends not only on total concentration but also on molecular form. Disulfide HMGB1 is particularly relevant because it is proinflammatory and has been linked to human liver ischemia–reperfusion injury and macrophage activation (103). Therefore, future biomarker studies should move beyond total HMGB1 quantification and incorporate redox-specific assays, kinetic profiling, and assessments of receptor activation (101, 103).

Circulating mtDNA is another high-priority biomarker candidate. Because mtDNA reflects mitochondrial disruption, an early postoperative increase in circulating mtDNA may detect reperfusion-sensitive bioenergetic injury before downstream complications are fully established (104). In liver transplantation, elevated circulating mtDNA has been associated with EAD, supporting its use as a mitochondrial injury readout. However, mtDNA interpretation requires standardized preanalytical handling, primer selection, normalization strategies, and sampling time points (104, 105).

cfDNA, donor-derived cfDNA (dd-cfDNA), extracellular histones, nucleosomes, and NET-associated markers provide complementary information. dd-cfDNA is particularly attractive because it can identify graft-specific injury in the recipient circulation (106–108). Extracellular histones and nucleosomes may better reflect chromatin toxicity, endothelial injury, and thromboinflammation (109–111). MPO–DNA complexes and CitH3 can indicate NET burden, but these markers should be interpreted selectively within a DAMP-centered framework rather than shifting the biomarker strategy solely to NETs (112, 113).

The major limitation of circulating DAMPs is their biological and technical heterogeneity (114). Their levels are influenced by ischemia time, graft type, surgical stress, infection, transfusion, renal clearance, hemolysis, immunosuppression, and systemic inflammation (115). Therefore, single DAMP measurements are unlikely to be sufficient for robust clinical interpretation. Their greatest clinical value will likely come from time-resolved panels that integrate source-specific DAMPs with conventional biomarkers and perioperative risk factors (116, 117).

### 6.2 Perfusate-accessible DAMPs during machine perfusion

Machine perfusion provides a distinctive opportunity for DAMP-based biomarker development (118). Unlike post-transplant blood sampling, perfusate sampling can be performed before implantation and can directly reflect graft-derived injury in a controlled ex vivo environment. This makes perfusate-accessible DAMPs especially relevant for marginal graft assessment, viability testing, and pre-reperfusion intervention (119, 120).

NMP maintains the graft in a metabolically active state and allows repeated assessment of lactate clearance, bile production, vascular resistance, aminotransferase release, oxygen consumption, and perfusate composition (121). HOPE provides mitochondrial support and has shown clinical benefit in reducing biliary complications in DCD grafts. These platforms are not merely preservation methods; they represent experimental and clinical windows into graft biology (122).

Perfusate DAMPs may improve the interpretation of machine-perfusion readouts. mtDNA, cfDNA, HMGB1, ATP-related signals, histones, extracellular vesicle-associated nucleic acids, and inflammatory proteins may indicate mitochondrial disruption, hepatocyte injury, endothelial stress, or biliary vulnerability before systemic reperfusion (114, 117). Recent studies assessing mtDNA or graft-derived cfDNA in perfusate and bile during ex vivo NMP support the feasibility of compartment-specific nucleic acid monitoring for graft-quality assessment. This approach is particularly relevant when conventional viability criteria are borderline (122, 123).

The major advantage and challenge of perfusate biomarkers is their graft specificity is the absence of standardized thresholds, respectively (101). Perfusate DAMP levels may vary according to perfusion duration, temperature, oxygenation strategy, perfusate composition, graft size, donor type, steatosis, warm ischemia time, and sampling protocol. Therefore, DAMP measurements during machine perfusion should be interpreted dynamically rather than as isolated absolute values. A rising mtDNA or cfDNA trajectory may be more informative than a single measurement (122).

### 6.3 DAMP levels and posttransplant complications

DAMPs may help connect early molecular injury with clinically relevant posttransplant outcomes (104, 105). EAD is a composite syndrome influenced by hepatocellular damage, mitochondrial failure, endothelial dysfunction, inflammation, and microcirculatory impairment. A DAMP profile enriched for HMGB1, mtDNA, cfDNA, histones, and inflammatory protein complexes may therefore provide a more biologically specific indicator of risk than aminotransferase levels alone (112).

Different DAMPs may correspond to different complications. mtDNA and oxidized mitochondrial products may indicate mitochondrial vulnerability and reperfusion sensitivity. HMGB1 may reflect inflammatory priming and macrophage activation (104). Extracellular histones and nucleosomes may signal endothelial toxicity and thromboinflammatory risk. S100A8/A9 and NET-associated markers may indicate sustained myeloid amplification (114, 115). Extracellular matrix-derived DAMPs may reflect sinusoidal injury, biliary stress, and later remodeling. This compartmental interpretation is essential because EAD, post-reperfusion syndrome, ischemic biliary complications, and rejection-associated injury may share overlapping biochemical features but differ in their dominant injury mechanisms (112).

Biliary complications deserve particular attention. Serum aminotransferase levels primarily reflect hepatocellular injury and may incompletely capture cholangiocyte vulnerability. Bile-derived or perfusate-derived DAMPs, especially nucleic acid signals and mitochondrial injury markers, may offer more direct insights into biliary epithelial stress and peribiliary microvascular injury. This is particularly important in DCD grafts, where ischemic cholangiopathy remains a major clinical challenge (119, 120).

DAMPs may also help identify patients who require closer monitoring or early intervention. For example, a patient with rapid normalization of aminotransferase levels but persistently elevated histone, cfDNA, or S100A8/A9 signals may still have ongoing endothelial or immune-mediated injury (112). Conversely, transient early DAMP release followed by rapid clearance may indicate reversible injury and adequate repair. Thus, both signal magnitude and clearance kinetics should be incorporated into risk models (123).

### 6.4 Detection technologies and assay standardization

The clinical translation of DAMP biomarkers depends on reliable detection technologies. Enzyme-linked immunosorbent assays (ELISAs) are widely used for protein-based DAMPs, including HMGB1, S100A8/A9, heat shock proteins, histones, and nucleosomes (101). However, many immunoassays do not distinguish distinct molecular forms, such as reduced versus disulfide HMGB1, free versus nucleosome-bound histones, or monomeric versus complexed S100 proteins. This limitation constrains mechanistic interpretation (102).

Quantitative polymerase chain reaction (qPCR) and digital polymerase chain reaction (dPCR) are commonly used for mtDNA and cfDNA detection. dPCR offers higher precision at low concentrations and may be particularly useful when DAMPs are measured in perfusate, bile, or early postoperative plasma (104). Next-generation sequencing (NGS) can provide tissue-of-origin information, fragmentomic features, methylation profiles, and dd-cfDNA quantification. These approaches may transform DAMP analysis from simple concentration measurement into injury-source mapping (107).

Proteomics, lipidomics, extracellular vesicle profiling, and mass spectrometry-based approaches may further expand DAMP detection (103). These methods are especially useful for identifying integrated injury signatures, including oxidized proteins, lipid peroxidation products, extracellular matrix fragments, and vesicle-associated nucleic acids. However, their current cost, analytical complexity, and turnaround time limit immediate routine clinical application (120, 121).

Standardization remains the central barrier. DAMP measurements are sensitive to sample type, anticoagulant choice, processing delay, centrifugation protocol, freeze–thaw cycles, hemolysis, DNA extraction method, primer design, assay calibration, and reporting units (111). For transplantation studies, additional variables include donor type, preservation method, ischemia time, reperfusion timing, immunosuppression, transfusion, and infection. Without standardized preanalytical and analytical workflows, DAMP cutoffs cannot be reliably compared across studies (123).

### 6.5 Toward multimarker DAMP panels

DAMP-based biomarker development is unlikely to depend on a single molecule. HIRI is a multicompartment injury process; therefore, biomarker panels should reflect hepatocellular, mitochondrial, endothelial, immune, biliary, and stromal aspects of injury (107). A rational DAMP panel might combine HMGB1 or HMGB1 isoforms for inflammatory priming, mtDNA for mitochondrial disruption, cfDNA or dd-cfDNA for graft injury, histones or nucleosomes for chromatin toxicity, S100A8/A9 or MPO–DNA complexes for myeloid amplification, and selected extracellular matrix fragments for remodeling or biliary risk (108).

Panel design should be time-specific. Preimplantation perfusate panels may focus on mtDNA, cfDNA, HMGB1, ATP-related signals, and endothelial injury markers. Early post-reperfusion plasma panels may prioritize mtDNA, HMGB1, histones, nucleosomes, and inflammatory cytokine-linked DAMPs (121). Later postoperative panels may incorporate S100A8/A9, NET-associated markers, extracellular matrix fragments, and bile-derived injury signals. This phased approach is more biologically plausible than applying the same panel across all time points (109).

DAMP panels should also be integrated with non-DAMP variables. The donor risk index (DRI), steatosis grade, cold and warm ischemia times, lactate clearance, perfusate aminotransferase levels, bile chemistry, oxygen consumption, vascular resistance, intraoperative hemodynamics, and postoperative liver function tests remain essential. DAMPs add value when they explain biological mechanisms that are not captured by these parameters (115–122).

For clinical implementation, a stepwise validation approach is most realistic. First, candidate DAMPs should be supported by mechanistic studies. Second, their kinetics should be tested in well-annotated liver transplantation and hepatic surgery cohorts (111). Third, multicenter studies should define sampling windows, thresholds, and outcome-specific panels. Finally, interventional trials should evaluate whether DAMP-guided decisions improve graft selection, preservation strategy, postoperative monitoring, or targeted therapy. In this framework, DAMPs become not only markers of injury but also tools for biomarker-guided clinical translation in HIRI (123).

## 7 Therapeutic and translational interfaces for DAMP-targeted intervention

### 7.1 Limiting DAMP release and preserving graft resilience

Therapeutic strategies targeting DAMPs in HIRI should be organized by their site of action within the injury cascade. The most upstream approach limits DAMP release at the source before extracellular amplification becomes self-sustaining (124, 125). This strategy requires maintaining hepatocyte membrane integrity, mitochondrial function, sinusoidal endothelial homeostasis, and redox balance during ischemia, preservation, implantation, and early reperfusion. Source-directed protection is particularly relevant because, once released, DAMPs can simultaneously activate multiple immune, vascular, and coagulation pathways (126).

Ischemic preconditioning (IPC), ischemic postconditioning (IPostC), and remote ischemic preconditioning (RIPC) are physiological approaches that reduce early DAMP spillover by improving stress tolerance before full reperfusion (121). Their protective mechanisms include stabilization of mitochondrial function, reduction of ROS generation, activation of endogenous cytoprotective pathways, improvement of sinusoidal perfusion, and attenuation of early inflammatory mediator release (104). These strategies are already familiar in hepatic surgery and transplantation, but their efficacy is variable and depends on graft type, steatosis, ischemia duration, operative setting, and recipient condition. Therefore, they should be considered broadly DAMP-modulating interventions rather than specific ligand-directed therapies (127).

Pharmacological source control has a more defined mechanistic basis. Mitochondria-targeted antioxidants, ferroptosis inhibitors, TRPM2 blockers, and ALOX12-directed strategies may reduce mtDNA release, lipid peroxidation, calcium-driven mitochondrial injury, and ROS-amplified DAMP generation (128). These approaches are most rational when applied before reperfusion or during the earliest reperfusion window, especially in steatotic, aged, or DCD grafts that have reduced mitochondrial reserve (129). However, most remain preclinical, and their clinical translation will require liver-directed delivery, careful safety assessment, and biomarker-based identification of grafts with dominant mitochondrial injury or lipid peroxidation signatures (130, 131).

### 7.2 Removing or neutralizing extracellular DAMP burden

Once DAMPs enter the extracellular space, therapeutic emphasis shifts from source control to DAMP removal or neutralization (132). This strategy is attractive because extracellular DAMPs act as receptor ligands, endothelial toxins, coagulation activators, and scaffolds for thromboinflammatory amplification. Compared with broad immunosuppression, extracellular neutralization aims to reduce danger burden while preserving selected downstream repair responses (80).

HMGB1 is the best-established extracellular target in experimental HIRI. Neutralizing HMGB1 or reducing its release attenuates hepatocellular injury, inflammatory cytokine production, and macrophage activation in preclinical models (73). A major translational challenge is that HMGB1 is not a single-function molecule. Its activity depends on redox state, post-translational modification, cellular source, and timing. Therefore, future strategies should distinguish pathogenic disulfide HMGB1 and HMGB1–TLR4/RAGE interactions from reparative or inactive forms of HMGB1 (80, 81).

Chromatin-directed neutralization represents another important therapeutic interface. Extracellular DNA, histones, nucleosomes, and NET-associated components can damage LSECs, activate platelets, promote fibrin deposition, and aggravate microvascular obstruction (133). Deoxyribonuclease-mediated degradation of extracellular DNA, histone neutralization, and inhibition of excessive NET formation may reduce this vascular-toxic DAMP burden. These approaches should be timed carefully because NETs and coagulation also contribute to antimicrobial defense and hemostasis (134).

A particularly innovative strategy involves multi-DAMP scavenging. The biosilica nanoparticulate scavenger, PEI-arg@MON@BA, was constructed through the biomimetic co-assembly of cfDNA-binding polyethylenimine (PEI), the nitric oxide substrate L-arginine (arg), reactive oxygen species (ROS)-scavenging tetrasulfur-bridged mesoporous organosilica nanoparticles (MONs), and the intracellular Ca²^+^ chelator BAPTA-AM (BA) (135). It was designed to scavenge cfDNA, ROS, and intracellular Ca²^+^ while supplying nitric oxide (NO); in preclinical HIRI and liver transplantation models, this platform reduced oxidative stress, cfDNA-induced TLR9–myeloid differentiation primary response 88 (MyD88)–NF-κB signaling, inflammatory cytokine production, and hepatic injury (135). This approach directly addresses a central limitation of single-target therapy: multiple DAMP classes are released simultaneously during reperfusion. It is therefore highly compatible with the multitarget logic summarized in **Table 2** (130, 135).

**Table 2:** Therapeutic strategies targeting damage-associated molecular pattern (DAMP)-mediated signaling pathways in hepatic ischemia–reperfusion injury.

Therapeutic strategy	Primary DAMP target or pathway	Mechanism of action	Evidence level	References
Therapeutics evaluated in animal/preclinical models				
HMGB1 inhibitors	HMGB1 → TLR4/RAGE signaling	Blocks HMGB1 release or receptor binding; reduces NF-κB activation	Preclinical rodent IRI models show reduced inflammation and hepatocellular injury	(21, 22)
TLR4 antagonists	HMGB1–TLR4 axis	Inhibits TLR4–MyD88 signaling; suppresses pro-inflammatory cytokine production	TAK-242 protective in murine liver IRI; Eritoran promising in sterile inflammation	(74–76)
RAGE inhibitors	HMGB1/S100A8/A9 → RAGE	Blocks ligand–RAGE interaction and MAPK amplification loop	Reduces ROS, MAPK activation, and neutrophil recruitment in preclinical studies	(79, 136)
NLRP3 inflammasome inhibitors	ATP/ROS/K^+^ efflux → NLRP3	Direct inhibition of NLRP3 assembly and caspase-1 activation	Markedly lowers IL-1β/IL-18 release and necroinflammation in liver IRI models	(82, 89)
P2X7 antagonists	ATP → P2X7	Blocks ATP-induced Ca²^+^ influx, pyroptosis, and inflammasome priming	Demonstrated protection in hepatic and renal IRI models	(25, 39)
TRPM2 blockers	ROS → TRPM2 channel	Inhibits Ca²^+^-dependent oxidative signaling and Kupffer cell activation	Reduces ROS-driven inflammation and hepatocyte death in experimental IRI	(5)
cGAS–STING pathway inhibitors	mtDNA → cGAS–STING	Blocks type-I IFN production and downstream inflammatory amplification	Shown to reduce Kupffer cell activation and tissue damage in murine IRI	(10, 139)
Ferroptosis inhibitors	Lipid peroxidation/ALOX12-12-HETE axis	Prevents iron-dependent lipid ROS accumulation	Strong protection in warm IRI models; decreases LSEC injury	(4, 5)
NETosis inhibitors	NET-derived DAMPs (DNA, histones)	Prevents NET formation or degrades extracellular DNA	Reduces microvascular obstruction and LSEC damage in IRI	(61–65)
S100A8/A9 neutralizing antibodies	S100A8/A9 → TLR4/RAGE	Blocks neutrophil–Kupffer cell inflammatory loop	Effective in sterile inflammation and IRI-related neutrophil activation	(31, 43)
Therapeutics applied or explored in clinical practice				
Antioxidants	ROS burst, mtDNA oxidation	Scavenges ROS; preserves mitochondrial integrity; reduces DAMP release	Clinically explored; strong preclinical evidence for reducing oxidative stress and DAMP release	(128, 129)
Ischemic preconditioning/remote ischemic preconditioning	Broad DAMP release modulation	Reduces mitochondrial rupture and dampens DAMP spillover	Validated in both hepatic surgery and transplantation studies	(124–127)
Machine perfusion–based interventions	Multiple DAMPs (HMGB1, ATP, mtDNA)	Enables graft-specific DAMP monitoring, viability assessment, and ex vivo intervention before implantation	Growing clinical evidence in liver transplantation perfusion systems	(13–15)

cGAS, cyclic GMP–AMP synthase; DAMP, damage-associated molecular pattern; HMGB1, high-mobility group box 1; IRI, ischemia–reperfusion injury; MyD88, myeloid differentiation primary response 88; NET, neutrophil extracellular trap; NLRP3, NLR family pyrin domain-containing 3; P2X7, P2X purinoceptor 7; RAGE, receptor for advanced glycation end products; ROS, reactive oxygen species; STING, stimulator of interferon genes; TLR4, Toll-like receptor 4; TRPM2, transient receptor potential melastatin 2.

### 7.3 Blocking dominant DAMP-sensing pathways

A third therapeutic approach involves blocking dominant DAMP-sensing pathways. This category includes TLR4 antagonists, RAGE inhibitors, P2X7 antagonists, NLRP3 inflammasome inhibitors, TRPM2 blockers, cGAS–STING inhibitors, ferroptosis inhibitors, NETosis inhibitors, and S100A8/A9-neutralizing strategies (75). These approaches constitute the largest preclinical category of interventions summarized in **Table 2** and are mechanistically promising but not yet clinically mature.

The HMGB1/S100A8/A9–TLR4/RAGE module is most relevant during inflammatory priming. TLR4 blockade with TAK-242 has shown protective effects in experimental and large-animal models of liver ischemia–reperfusion, supporting the translational potential of DAMP-sensor inhibition (76). Strategies targeting RAGE may reduce HMGB1- and S100-driven MAPK signaling, oxidative stress, neutrophil recruitment, and endothelial activation. However, systemic inhibition of these pathways may interfere with host defense and repair; therefore, transient perireperfusion blockade or graft-localized delivery is preferable (136).

The ATP–P2X7–NLRP3 axis represents a second targetable pathway. P2X7 antagonists and NLRP3 inhibitors can suppress potassium efflux, inflammasome assembly, caspase-1 activation, IL-1β maturation, and pyroptotic DAMP propagation in experimental ischemia–reperfusion models (137). These interventions are most appropriate during early reperfusion and early inflammatory propagation. Prolonged systemic inflammasome inhibition, however, may impair antimicrobial immunity and debris clearance, making short-course or ex vivo application more attractive (138).

The mtDNA–cGAS–STING axis is an emerging target in nucleic acid sensing. Experimental work indicates that blocking mtDNA release or inhibiting cGAS–STING signaling reduces inflammatory activation and tissue injury in hepatic ischemia–reperfusion models. This pathway may be especially relevant in grafts with pronounced signatures of mitochondrial injury in perfusate or early postoperative plasma (136). Nevertheless, because DNA sensing contributes to antiviral defense and immune surveillance, clinical translation will require precise timing, compartment-specific delivery, and careful risk stratification (139).

Ferroptosis and calcium-dependent oxidative injury also offer important therapeutic avenues. TRPM2-mediated calcium influx can increase ALOX12-dependent lipid peroxidation and ferroptotic injury, whereas ferroptosis inhibitors may reduce lipid ROS accumulation and downstream DAMP release (131). These strategies are particularly relevant in metabolically vulnerable grafts, including fatty livers, in which lipid substrates and impaired mitochondrial reserve amplify reperfusion injury (130–132).

### 7.4 Machine perfusion as a translational platform

Machine perfusion offers the most practical bridge between DAMP biology and clinical translation. NMP maintains graft metabolism and enables functional assessment before implantation, whereas HOPE supports mitochondrial recovery and reduces the risk of biliary injury in DCD grafts (140). These approaches should not be viewed merely as preservation technologies. Rather, they provide a controlled ex vivo window for DAMP monitoring, graft assessment, and therapeutic intervention (141).

From a DAMP-centered perspective, machine perfusion has three major advantages. First, perfusate sampling can detect graft-derived DAMPs before recipient systemic inflammation confounds interpretation (140). Second, repeated measurements can reveal DAMP trajectories rather than isolated values. Third, therapeutic agents can be delivered directly to the graft, potentially reducing systemic exposure. For example, a graft with high perfusate mtDNA and poor lactate clearance may require mitochondrial stabilization or DNA-sensing pathway modulation, whereas a graft with high chromatin, histone, or endothelial injury markers may benefit more from extracellular DAMP neutralization or endothelial protection (38).

Machine perfusion is also well suited for testing the therapies summarized in **Table 2**. Antioxidants, extracellular DNA degradation, HMGB1 neutralization, multi-DAMP scavengers, NLRP3 inhibition, cGAS–STING modulation, endothelial protectants, and mesenchymal stem/stromal cell-derived extracellular vesicles (MSC-EVs) could be evaluated ex vivo before systemic clinical administration (135). This is especially important for interventions with potential risks of infection, coagulation disturbance, or impaired regeneration (140, 141).

### 7.5 Cell-, vesicle-, and biomarker-guided combination strategies

Cell- and vesicle-based therapies provide broader immunoregulatory strategies. Mesenchymal stem/stromal cells (MSCs) and MSC-derived extracellular vesicles (MSC-EVs) can reduce oxidative stress, suppress inflammatory cytokine production, modulate macrophage polarization, promote mitochondrial quality control, and support tissue repair in preclinical HIRI models (142). Their strength lies in multimodal activity rather than single-ligand blockade. However, vesicle heterogeneity, manufacturing standards, dosing, biodistribution, storage, and potency assays remain major barriers. At present, MSCs and MSC-EVs are best viewed as promising preclinical modulators rather than established clinical therapies (143, 144).

The complexity of DAMP biology makes single-node therapy unlikely to succeed across all grafts and recipients. A more realistic strategy is stage-specific and compartment-specific combination therapy (145). During preservation and preimplantation, treatment should prioritize mitochondrial stabilization, endothelial protection, and reduction of early DAMP release (146). During early reperfusion, transient blockade of HMGB1/TLR4/RAGE, mtDNA/cGAS–STING, or ATP/NLRP3 signaling may reduce inflammatory priming (147). During delayed reperfusion, extracellular DNA, histones, NET-associated DAMPs, platelet–endothelial interactions, and thromboinflammation become more actionable. During repair, excessive suppression may be harmful; therapy should instead favor DAMP clearance, immune resolution, cholangiocyte protection, and matrix homeostasis (148).

Clinical translation should therefore proceed stepwise. First, DAMP assays and sampling windows must be standardized (149). Second, DAMP panels should be validated in multicenter liver transplantation and hepatic surgery cohorts. Third, DAMP signatures should be integrated with donor risk, ischemia time, steatosis, lactate clearance, bile chemistry, perfusion parameters, aminotransferase levels, and postoperative outcomes (101). Finally, interventional trials should specifically target high-risk grafts or recipients with defined DAMP signatures. In this framework, **Table 2** should be interpreted not as a list of isolated drugs but as a map of therapeutic interfaces: preclinical agents define mechanistic opportunities, whereas antioxidants, ischemic conditioning, and machine perfusion represent the most clinically accessible platforms for near-term translation (140, 141).

**Figure 3:**
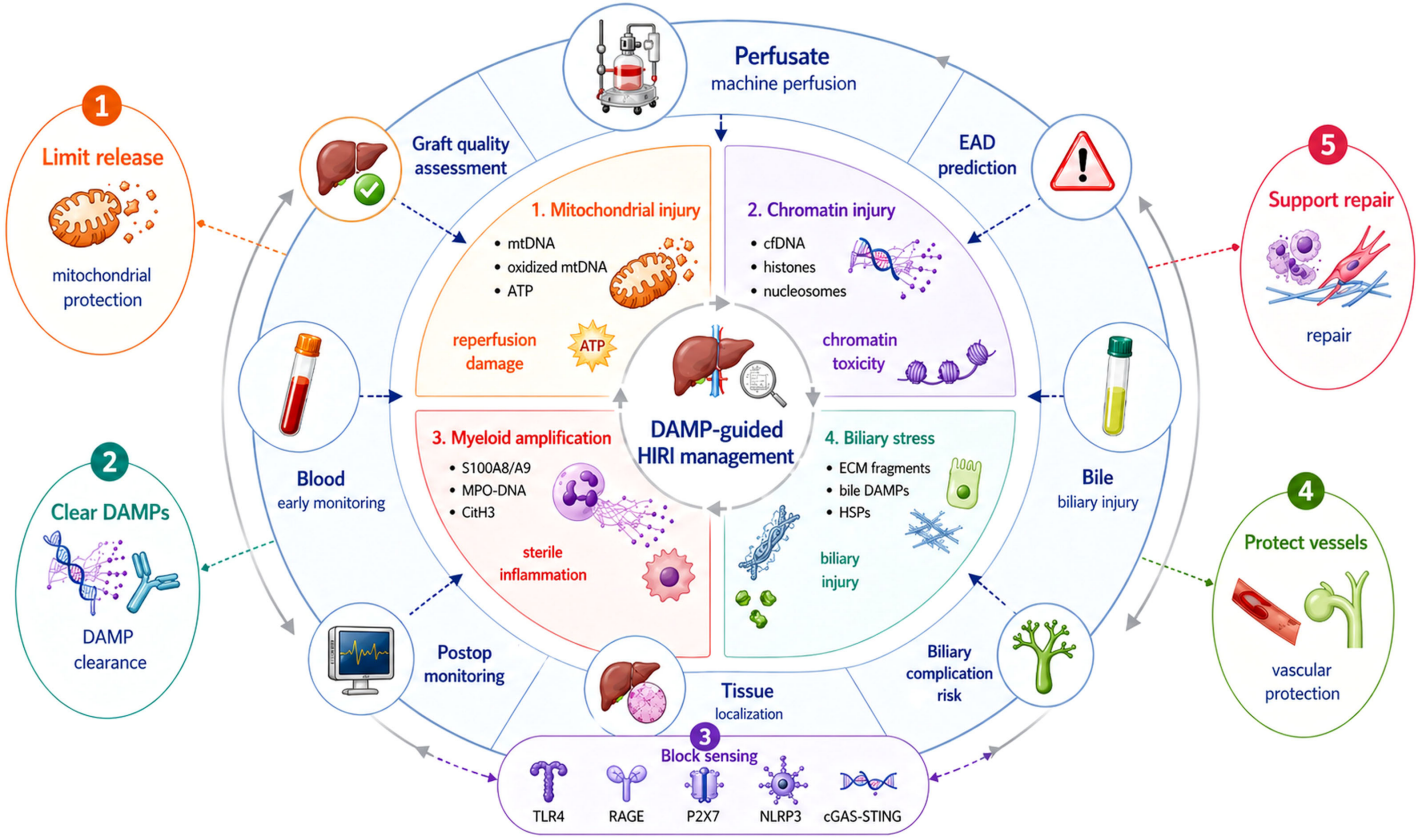
Biomarker-guided clinical translation and therapeutic interfaces for DAMP-centered HIRI management. Damage-associated molecular patterns (DAMPs) detected in perfusate, blood, bile, and tissue can be integrated into injury-specific signatures reflecting mitochondrial injury, chromatin toxicity, myeloid amplification, and biliary stress during hepatic ischemia–reperfusion injury (HIRI). These signatures support graft-quality assessment, early allograft dysfunction (EAD) prediction, biliary complication risk stratification, postoperative monitoring, and targeted interventions that limit DAMP release, enhance clearance, block sensing pathways, protect vascular compartments, and support repair.

Overall, DAMP-targeted therapy should move beyond a “one molecule–one pathway” model. The goal is not to abolish sterile inflammation, because controlled DAMP signaling may support debris clearance, host defense, and repair (150). A more realistic objective is to limit excessive DAMP release, neutralize pathogenic extracellular DAMPs, block dominant maladaptive sensing pathways, and preserve repair-competent responses. This biomarker-guided, timing-specific approach is more consistent with the spatiotemporal biology of HIRI and more suitable for translation into liver transplantation and hepatic surgery (**Figure 3**) (151).

## 8 Discussion and future directions

Future research on DAMPs in HIRI should move from single-molecule descriptions toward integrated, time-resolved, and compartment-specific models (152). Current evidence supports the importance of HMGB1, mtDNA, cfDNA, extracellular histones, S100A8/A9, and extracellular matrix-derived fragments. However, their biological meaning depends strongly on cellular source, molecular form, release timing, and sampling compartment (153, 154). Therefore, the next priority is not simply to identify additional DAMPs, but to define how specific DAMP signatures emerge across cold ischemia, warm ischemia, early reperfusion, delayed inflammatory amplification, and repair or remodeling (155, 156).

A key direction is the construction of spatiotemporal DAMP atlases. Single-cell transcriptomics, spatial transcriptomics, proteomics, metabolomics, extracellular vesicle profiling, and mass spectrometry imaging can help define which cell populations generate or respond to DAMPs at each stage of HIRI. Such atlases should link DAMP release to anatomical zones, immune-cell localization, endothelial injury, biliary vulnerability, and fibrotic remodeling (157). This approach would help distinguish initiating DAMPs from downstream amplification signals and repair-associated mediators (158).

Another urgent need is to establish clinically useful sampling windows. DAMP levels may have different meanings before implantation, during machine perfusion, immediately after reperfusion, within the first postoperative day, or during delayed graft recovery. Preimplantation perfusate measurements may identify graft vulnerability before systemic reperfusion, whereas early postoperative blood measurements may capture mitochondrial rupture, chromatin toxicity, or inflammatory priming (159). Later measurements may better reflect persistent endothelial injury, biliary stress, or remodeling. Therefore, future studies should prioritize DAMP kinetics and clearance patterns rather than isolated single-time-point values (160, 161).

Multicenter validation is essential before DAMPs can be incorporated into clinical decision-making. Existing studies remain limited by heterogeneous sampling protocols, assay platforms, preservation methods, and outcome definitions (162). Prospective cohorts should harmonize donor classification, ischemia-time reporting, machine-perfusion parameters, sample processing, and endpoints such as EAD, post-reperfusion syndrome, ischemic biliary complications, rejection, graft survival, and hospitalization (163). DAMP panels should be tested for additive value beyond established clinical variables, including donor risk, steatosis grade, lactate clearance, perfusate aminotransferase levels, bile chemistry, vascular resistance, oxygen consumption, and postoperative liver function tests (164).

Therapeutic translation should also become more selective. Because DAMP signaling can support debris clearance, host defense, immune resolution, and tissue repair, broad suppression is unlikely to be optimal (165). Instead, therapy should target the dominant pathogenic DAMP module within a defined time window. During preservation or machine perfusion, strategies may focus on mitochondrial stabilization, endothelial protection, and extracellular DAMP removal (159, 166). During early reperfusion, transient blockade of inflammatory sensing pathways may reduce injury amplification. During delayed injury, extracellular DNA, histones, NET-associated DAMPs, and thromboinflammation may become more relevant targets. During repair, treatment should favor DAMP clearance, cholangiocyte protection, and matrix homeostasis (152, 167).

Machine perfusion is likely to be the most practical platform for implementing these advances. It enables repeated DAMP monitoring, *ex vivo* therapeutic delivery, dynamic graft assessment, and reduced systemic exposure (158). Ultimately, DAMP-centered research should integrate molecular signatures with perfusion parameters and clinical risk models, shifting HIRI management from delayed injury recognition toward proactive graft evaluation and biomarker-guided intervention (168).

## 9 Conclusion

HIRI is a dynamic, multicompartment process involving metabolic failure, mitochondrial disruption, endothelial injury, immune-cell activation, thromboinflammation, biliary vulnerability, and repair-associated remodeling (53). DAMPs provide a unifying framework for understanding this complexity. Rather than only serving as upstream inflammatory triggers, they function as spatiotemporal injury signatures that indicate where injury begins, when it evolves, which cellular compartments are involved, and which downstream pathways become dominant (23, 59).

This review reframes DAMP biology according to source, timing, sensing pathway, sampling compartment, and intervention window. Nuclear and nucleic acid-associated DAMPs indicate chromatin and hepatocellular injury; mitochondrial and metabolic DAMPs reflect reperfusion-sensitive bioenergetic failure; extracellular matrix-derived and protein-based DAMPs capture vascular, biliary, stromal, and remodeling responses; and DAMP complexes, including NET-associated components, amplify thromboinflammation and microvascular dysfunction. This integrated view moves the field beyond isolated inflammatory pathways toward a more clinically useful model of injury progression.

The strongest near-term translational potential lies in biomarker development and graft assessment. Circulating DAMPs may support postoperative risk stratification, whereas perfusate- and bile-accessible DAMPs may help evaluate graft quality before implantation, particularly during machine perfusion (159). However, single-analyte approaches are unlikely to capture the full complexity of HIRI. Future implementation will require standardized assays, clearly defined sampling windows, multicenter validation, and multimarker panels integrated with donor features, perfusion parameters, conventional liver tests, and clinical outcomes (169).

Therapeutically, DAMP-centered strategies should not aim to indiscriminately abolish sterile inflammation. A more realistic goal is to limit excessive DAMP release, neutralize pathogenic extracellular DAMP burden, block dominant maladaptive sensing pathways, and preserve repair-competent responses (124, 170). If these challenges are addressed, DAMP-centered analysis may support proactive graft protection, safer use of marginal organs, and biomarker-guided precision intervention in liver transplantation and hepatic surgery (152, 171).

## Glossary

HIRI: hepatic ischemia–reperfusion injury
EAD: early allograft dysfunction
DAMPs: damage-associated molecular patterns
PRRs: pattern-recognition receptors
TLRs: Toll-like receptors
RAGE: receptor for advanced glycation end products
P2X7: P2X purinoceptor 7
NLRP3: NLR family pyrin domain-containing 3
cGAS: cyclic GMP–AMP synthase
STING: stimulator of interferon genes
DCD: donation after circulatory death
ATP: adenosine triphosphate
LSECs: liver sinusoidal endothelial cells
KCs: Kupffer cells
ROS: reactive oxygen species
HMGB1: high-mobility group box 1
cfDNA: cell-free DNA
mtDNA: mitochondrial DNA
NF-κB: nuclear factor kappa B
MAPK: mitogen-activated protein kinase
NETs: neutrophil extracellular traps
IRI: ischemia–reperfusion injury
NMP: normothermic machine perfusion
HOPE: hypothermic oxygenated machine perfusion
NET-DNA: neutrophil extracellular trap-associated DNA
S100A8/A9: S100 calcium-binding protein A8/A9
MPO-DNA: myeloperoxidase-DNA complexes
CitH3: citrullinated histone H3
ALOX12: arachidonate 12-lipoxygenase
12-HETE: 12-hydroxyeicosatetraenoic acid
ECM: extracellular matrix
TLR4: Toll-like receptor 4
TNF-α: tumor necrosis factor alpha
TLR9: Toll-like receptor 9
HSPA12A: heat shock protein family A member 12A
IL-1β: interleukin-1 beta
IL-6: interleukin-6
TRPM2: transient receptor potential melastatin 2
NO: nitric oxide
vWF: von Willebrand factor
MPO: myeloperoxidase
PAD4: peptidylarginine deiminase 4
DNase I: deoxyribonuclease I
NOX2: nicotinamide adenine dinucleotide phosphate oxidase 2
HSCs: hepatic stellate cells
YAP: Yes-associated protein
TAZ: transcriptional coactivator with PDZ-binding motif
S100A9: S100 calcium-binding protein A9
TLR2: Toll-like receptor 2
IL-18: interleukin-18
GSDMD: gasdermin D
caspase-1: cysteine-aspartic protease-1
CpG: cytosine-phosphate-guanine
TBK1: TANK-binding kinase 1
IRF3: interferon regulatory factor 3
ALT: alanine aminotransferase
AST: aspartate aminotransferase
INR: international normalized ratio
dd-cfDNA: donor-derived cell-free DNA
ELISAs: enzyme-linked immunosorbent assays
qPCR: quantitative polymerase chain reaction
dPCR: digital polymerase chain reaction
NGS: next-generation sequencing
DRI: donor risk index
IPC: ischemic preconditioning
IPostC: ischemic postconditioning
RIPC: remote ischemic preconditioning
PEI-arg@MON@BA: PEI/arginine/MON/BAPTA-AM co-assembled biosilica nanoparticulate scavenger
PEI-arg@MON@BA: a biosilica nanoparticulate scavenger co-assembled from polyethylenimine, L-arginine, tetrasulfur-bridged mesoporous organosilica nanoparticles, and BAPTA-AM
MyD88: myeloid differentiation primary response 88
MSCs: mesenchymal stem/stromal cells
MSC-EVs: mesenchymal stem/stromal cell-derived extracellular vesicles
